# Survivorship of geographic *Pomacea canaliculata* populations in responses to cold acclimation

**DOI:** 10.1002/ece3.6162

**Published:** 2020-03-23

**Authors:** Zhong Qin, Rui Shan Wu, Jiaen Zhang, Zhi Xin Deng, Chun Xia Zhang, Jing Guo

**Affiliations:** ^1^ The Department of Ecology College of Natural Resources and Environment South China Agricultural University Guangzhou China; ^2^ Guangdong Provincial Key Laboratory of Eco‐Circular Agriculture Guangzhou China; ^3^ Guangdong Engineering Technology Research Centre of Modern Eco‐agriculture and Circular Agriculture Guangzhou China; ^4^ Key Laboratory of Agro‐Environment in the Tropics Ministry of Agriculture and Rural Affairs Guangzhou China; ^5^ Henry Fok College of Life Sciences Shaoguan University Shaoguan China

**Keywords:** apple snails, body size, cold acclimation, geographic location, survival

## Abstract

1. *Pomacea canaliculata*, a freshwater snail from South America, has rapidly established natural populations from south to north subtropical region in China, since its original introductions in the 1980s. Low temperature in winter is a limiting factor in the geographic expansion and successfully establishment for apple snail populations. There have been some studies on population level of low temperature tolerance for *P. canaliculata*, yet little is quantified about its life‐history traits in responses to cold temperatures. Whether these responses vary with the acclimation location is also unclear. We investigated the survivorship and longevity of *P. canaliculata* in responses to cold temperatures and examine whether these responses vary with the location and snail size. We hypothesized that survival of the snails depends on their shell height and the level of low temperature, and *P. canaliculata* population from the mid-subtropical zone may exhibit the highest viability over the cold thermal range.
2. We sampled *P. canaliculata* populations from five latitude and longitude ranges of subtropical China: Guangzhou population in southernmost (SM‐GZ), three populations of Yingtan (MR‐YT), Ningbo (MR‐NB), Ya'an (MR‐YA) in midrange, and Huanggang population in northernmost (NM‐HG) subtropical zone. For each *P. canaliculata* population, survival and longevity at six cold acclimation temperature levels (12, 9, 6, 3, 0, and −3°C) were quantified, and the effects of location and shell height were examined.
3. The MR‐YA population from mid-subtropical zone of China exhibited the highest survival rate and prolonged survival time regardless of the temperature acclimation treatments, whereas the SM‐GZ population from southern subtropical was the most sensitive to cold temperatures, particular temperatures below 9°C. No individuals of the SM‐GZ population could survive after stressed for 30 days (3°C), 5 days (0°C) and 2 days (−3°C), respectively. For each experimental *P. canaliculata* population held at 3, 0, and −3°C, individuals with intermediate shell height of 15.0–25.0 mm had significantly higher survivals.
4. The results highlight a request of a more thorough investigation on acclimation responses in each of the life table demographic parameters for *P. canaliculata*, and pose the question of whether natural selection or some genetic changes may have facilitated adaptation in invasive locations.

## Graphical abstract

Survival of *Pomacea canaliculata* depends on their shell height and the level of low temperature. *P. canaliculata* population from the mid‐subtropical zone exhibit the highest viability over the cold thermal range.

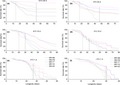

## 1 INTRODUCTION

*Pomacea canaliculata* (Lamarck) (Gastropoda: Ampullariidae), a freshwater snail native to tropical and temperate South America, has become a severe invasive agricultural pest in many Asian countries including the Philippines, Vietnam, Thailand, Japan, and Korea (Hayes et al., 2015). This species was notorious due to the great damage to indigenous aquatic crops (especially commercial rice) (Joshi, Cowie, & Sebastian, 2017; Smith & Fowler, 2002), severe impacts to various water environments (Carlsson, Brönmark, & Hansson, 2004; Horgan, Stuart, & Kudavidanage, 2014) and threats to public health as vectors of a parasitic nematode (Kim, Hayes, Yeung, & Cowie, 2014) and perhaps some trematodes (Kim et al., 2014).

Temperature plays a key determinant of activity thresholds (Stevens, Welch, Darby, & Percival, 2002), growth (Ito, 2002), reproduction (Estebenet & Martín, 2002), development (Seuffert, Burela, & Martín, 2010), and survival (Matsukura & Wada, 2007) of *P. canaliculata*. *Pomacea canaliculata* are adaptable to various temperature conditions. The ability to survive at low temperature constitutes a critical factor for successful range expansion of *P. canaliculata* in temperate East Asia as well as tropical Southeast Asia (Ito, 2002; Yoshida, Matsukura, Cazzaniga, & Wada, 2014). Low temperature acclimation involves a large suite of molecular, biochemical, and physiological adjustments. Evidence has shown that *P. canaliculata* can tolerate cold conditions during winter and that they have developed a range of physiological and behavioral strategies that promote overwinter survival and establishment in temperate East Asia (Bae & Park, 2015; Matsukura, Tsumuki, Izumi, & Wada, 2008, 2009). Cold tolerance of *P. canaliculata* links to distribution limits and might therefore be used to predict responses to future climate change. The southern/northern distribution limits of *P. canaliculata* was estimated through studies of its cold tolerance, including supercooling points (SCPs), survival under low temperatures, seasonal adaption assessment, and acclimation efficiency (Byers et al., 2013; Park, Bae, & Kwon, 2012; Seuffert et al., 2010). *Pomacea canaliculata* may increase its cold hardiness after cold acclimation. A comparative study indicated that tropical snails from the Philippines and snails originating from temperate Japan showed enhanced cold hardiness after cold acclimation, though the degree of cold hardiness was similar among the different populations (Wada & Matsukura, 2011). Compared with various studies of *P. canaliculata* in temperate and tropical regions, we know less about the adaption and survivors of cold‐tolerant snails from subtropical populations.

*Pomacea canaliculata* was intentionally introduced to Zhongshan, Guangdong province, China, in the early 1980s, and its range has extended from the Pearl River valley northwards to the Yangtze River basin, covering a broad range of thermal and seasonal habitats in 17 provinces including Hainan, Guangdong, Guangxi, Fujian, Zhejiang, Jiangxi, Jiangsu, Shanghai, Anhui, Hubei, etc. (Yang, Liu, He, & Yu, 2018). It should be noted that another highly invasive apple snail species, *Pomacea maculata* (Perry, 1,810), was also introduced and established in China since 1980s (Yang et al., 2018). Because the two apple snail species were difficult to differentiate morphologically from each other, *P. canaliculata* was presumed to be the only non‐native apple snail species in China, until recent molecular phylogenetic data were successfully used to separate the two closely related congenerics (Hayes, Cowie, Thiengo, & Strong, 2012). In this study, only *P. canaliculata* was used for testing response to cold acclimation.

Low temperatures during winter play an important role in limiting the distribution and reproduction of *P. canaliculata* in south China, where the weather is characterized as a subtropical monsoon climate with alternating moist and dry seasons (Xie & Su, 2014). Under natural conditions, *P. canaliculata* generally developed in one or two generations per year in southeast provinces such as Zhejiang, Jiangxi, and Sichuan (Zhou, Wu, & Yang, 2003). Liu and colleagues (Liu et al., 2014) observed the snails to hibernate in paddy fields, irrigation canals, or ponds in early November, until the fields were irrigated for rice planting in early April of the next year. *Pomacea canaliculata* could develop three generations per year in further southern provinces of Hainan and Guangdong. The snails tend to stay motionless, safely overwintering with low mortality in natural environments (Zhou et al., 2003). In Hainan, the snails can stay active throughout the year under mild and frost‐free conditions, whereas in Guangdong, the snails can safely overwinter in natural environments by hiding themselves in the topsoil and slowing down their metabolism (Zhou et al., 2003). Field observation also showed that snails in Guangdong usually exhibited a rather quick response to instantaneous temperature changes, being able to recover quickly and to continue development once the conditions were favorable (He et al., 2011).

Given the diverse thermal conditions *P. canaliculata* encountered during its dispersal from southern to north subtropical region, this species could be an ideal model organism for investigating the divergence in ecological requirements and cold adaptation among geographic populations. Several studies have examined the population differences in life‐history traits including shell height, egg features, development time, and importantly, heat and cold tolerance (Xu, Han, & Zhang, 2009; Xu et al., 2015, 2016). The heat and cold tolerance of five geographic populations of *P. canaliculata* inhabiting southern China exhibited opposing latitudinal clines, in which Nanning population (22.82°N, 108.37°E) had the strongest heat tolerance and the weakest cold tolerance, while Kumming population (25.05°N, 102.72°E) had the lowest heat tolerance and highest cold toleranc (Dong, Bai, Pan, & Yu, 2010). These clinical studies provide evidence that *P. canaliculata* populations differ in their ability to counter thermal stresses, and they underscore the importance of the investigation of both physiological and ecological factors contributing to the evolution of thermotolerance in nature. However, little information exists on population differences because most studies use individuals in experiments. Studies on the increase of cold hardiness after cold acclimation in *P. canaliculata* populations are also limited.

A size‐dependent effect of cold tolerance in *P. canaliculata* has been shown in both field survey and laboratory studies. The body size of snails (i.e., shell height) and its combined effect with the other factors (i.e., water conditions and sex) were reported to affect their survival during winter dormancy. Juveniles with shell height around 10 mm can overwinter in drained paddy fields, whereas adults can overwinter only at flooded sites because of their insufficient cold tolerance under dry conditions (Wada & Matsukura, 2007; Yoshida, Hoshikawa, Wada, & Yusa, 2009). The effects of water conditions (dry or moist) affected the survival of the snails through interactions with body size and duration (Yusa, Wada, & Takahashi, 2006). The juvenile strategy seemed to be to assure survivorship with very low growth rates under low temperatures (Seuffert & Martin, 2013). Therefore, individual snail size data are essential and should be included in elucidating interpopulation differences in cold tolerance.

We quantified cold tolerance in terms of survival rate and longevity for five distinct geographic populations of *P. canaliculata*, with the aim to determine how responses to winter cold temperatures vary with location and snail size. We hypothesized that snail survival depended on their shell height and low temperature, and we further predicted that the *P. canaliculata* population from the mid‐subtropical zone would exhibit the highest viability over the cold thermal range. Because the sampled *P. canaliculata* populations span the current dispersal range of *P. canaliculata* in southernChina, results of this laboratory work could be helpful to deductively forecast the regional winter field mortality in similar types of habitats, and it may extend the understanding of mechanisms of thermal adaptation in diverse subtropical climates.

## 2 METHODS

### 2.1 Field sampling and study populations

**FIGURE 1:**
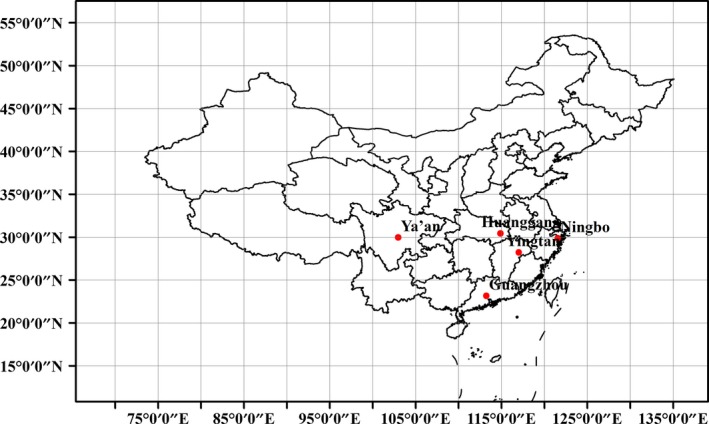
Five sampling locations (Ningbo, Huanggang, Yingtan, Ya'an, and Guangzhou) for *Pomacea canaliculata*

Populations of *P. canaliculata* were collected respectively in five locations (Figure 1) representing different thermal habitats along the Yangtze River Valley and its southern parts (see Table 1 for geographic and climate details of all populations). In this area, paddy fields are irrigated and rice is transplanted in mid‐ or late June/July. The paddy water is retained when rice harvesting concludes at the end of October to early November. We collected snails visibly out of their shell or attached to substrate from submerged paddy fields in early August 2014, wrapped them in moist paper towels, placed them in a cooler with ice and transported them immediately to the laboratory in the Ecology department of South China Agricultural University, where the experiments described below were conducted for each collected population. All the testing snails sampled from paddy fields were discerned using mitochondrial DNA (COI) analysis (Yang et al., 2018) to make sure that only *P. canaliculata* species will be used for the low temperature acclimation experiments.

**TABLE 1:** Geographic, temperature and precipitation data for five *Pomacea canaliculata* populations sampled across southern China

Location	Population abbreviation	Latitude (N)	Longitude (E)	Ann.Temperature (°C)	Jan.Tmin (°C)	Jan.Tax (°C)	July.Tmax (°C)	Ann.Precipitation (mm)	Altitude (Meters)
Guangzhou, Guangdong	SM‐GZ	23°10′7.62″	113°21′45.34″	22.1	10.3	18.3	28.7	1,982.7	14
Yingtan, Jiangxi	MR‐YT	28°12′47.88″	116°48′41.81″	18.4	3.4	10.1	29.7	1,750.0	36
Ningbo, Zhejiang	MR‐NB	29°49′32.91″	121°39′35.65″	16.7	1.6	8.0	28.0	1,480.0	6
Ya′an, Sichuan	MR‐YA	29°59′5.63″	103°4′31.83″	16.3	4.1	9.4	22.0	1,706.0	552
Huanggang, Hubei	NM‐HG	30°47′33.24″	115°43′11.67″	17.5	0.4	9.0	33.4	1,098.2	124

Note

Climate data (monthly standard values for the period 1971–2000) were obtained from Chinese Ecosystem Research Network dataset (http://www.cern.ac.cn/0index/). Jan.Tmin/Tmax, average minimum/maximum temperature in January; Ann.Temperature, annual mean temperature; Ann.Precipitation, annual mean precipitation.

*P. canaliculata* populations were sampled from five latitude and longitude ranges of subtropical China: Guangzhou population in southernmost (SM‐GZ), three populations of Yingtan (MR‐YT), Ningbo (MR‐NB), Ya'an (MR‐YA) in midrange, and Huanggang population in northernmost (NM‐HG) subtropical zone.

Samples of *P. canaliculata* were reared separately in a 135‐L rectangular aquarium (70 cm long × 52 cm wide × 37 cm high) filled with water to a depth of 15 cm at 25 ± 1°C and a photoperiod L10: D14. Sufficient fresh lettuce (*Lactuca sativa*) were provided every day, and the remains were carefully removed the next day at the same time. For each population, three size groups of snails were randomly selected and marked with paint ten days before their use in the experiments: small juveniles with a shell height of 7.5–12.5 mm (H1), juveniles with a shell height of 15.0–25.0 mm (H2), and adults of 25.0–35.0 mm (H3). To ensure the proper grouping, each juvenile was identified for the sex (when possible) based on its operculum morphology and by checking through the shell for a visible albumin gland or testes (Cazzaniga, 1990).

A total of 30 individuals with three size groups (each group included 10 snails) were pooled and arranged into a new aquarium (27 cm long × 15 cm wide × 16 cm high) to be used for cold tolerance assessment. Aerated water was introduced into each aquarium and maintained at a depth of 15 cm, and water was continuously aerated to maintain a 15 cm depth. Throughout the experiment, each aquarium was cleaned and the water was changed every 2 days. Egg masses were checked daily and recorded if possible. Sex of *P. canaliculata* was excluded from the temperature acclimation experiments because we did not separate sexes as we could not reliably sex all snails.

### 2.2 Low temperature acclimation experiments

The temperature experiment was designed to characterize the impact of typical winter conditions within and beyond the current distribution range of *P. canaliculata* in China as naturally as possible. Six low temperature acclimation regimes (12, 9, 6, 3, 0, and −3°C) were chosen based on the 30‐year average January temperature regimes of the five sample locations (see Table 1). The lowest temperature level (−3°C) corresponded to extreme winter conditions in the northern geographic range margin of *P. canaliculata*. For each temperature acclimation regime, *P. canaliculata* populations (with three size group individuals mixed) reared in aquaria respectively were stored in controlled climate chambers (made by Beijing Chance International Instrument Co. Ltd.) and were randomly assigned to one of the temperature treatments under a L10: D14 photoperiod. Starting from 25°C, the temperature was changed at a rate of 1°C per day until the desired temperature was reached, and then, this test temperature was held to check for survivorship. Three replicates were conducted for each *P. canaliculata* population and internal size group combination. The snails were observed closely for activity or limb movement every day. Survival was scored for all treatments until no change in mortality for five consecutive days or mortality reached 100%. The survival rate (100 × number of live snails/ total number of snails) at the end of the experiment and the mean survival time were calculated subsequently.

### 2.3 Statistical analysis

Laboratory cold acclimation tests of *P. canaliculata* populations were conducted for 60 days. To assess responses to different cold acclimation temperatures, survival of populations after a certain exposure time was set for the temperature treatments and used for statistical analysis: T1 (12 and 9°C, exposed for 30 days), T2 (6 and 3°C, exposed for 10 days), and T3 (0 and −3°C, exposed for 1 day). The derived datasets for all experimental *P. canaliculata* groups were assessed for normality using a Shapiro–Wilk test before analyses. Box‐Cox analysis was employed to meet the requirements of normality when necessary. Two impact factors on survivorship of *P. canaliculata* under the specific acclimation temperature were considered: geographic location (5 levels) and shell height (3 levels), and results were analyzed with two‐way ANOVA followed by the Fisher's least significant difference (at the 0.05 level) for post hoc comparisons. To describe survivorship of *P. canaliculata* populations as a continuous function of temperature, survival rates (following a 1 day exposure) at different temperatures for each population were explored through nonlinear regression analysis. To test whether survival of the snail was related to geographic location, temperature and body size, the Cox proportional hazards regression model (Cox, 1992) with a likelihood‐ratio test was employed, using replicates as random effect. For any given combination of population–temperature–size trial, the differences in the proportion of snail surviving between treatment boxes and their corresponding controls (i.e., the SM‐GZ population, corresponding to the fifth sampling location; at temperature of 12°C, corresponding to the highest temperature acclimation regime, and snail in H1 group, corresponding to small juveniles with shell height of 7.5–12.5 mm) were further compared. In these cases, Bonferroni corrections were used to adjust the *p* value for multiple tests. All statistical analyses were performed using SPSS 22.0 (SPSS Inc.).

## 3 RESULTS

### 3.1 Survival and longevity

**FIGURE 2:**
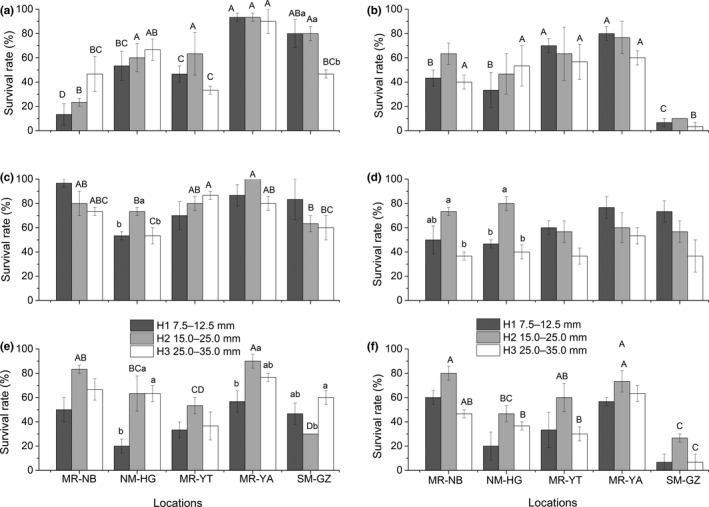
Survivorship of *Pomacea canaliculata* populations with different shell height at each experimental acclimation temperature. Mean survival and standard errors (*n* = 3) for each population were also shown. Under the specific temperature, different capital letters indicate significant differences in survival rate between locations for each size group, while small letters indicate significant differences between size groups for each location (*p* < .05). The three size groups of snails were as follows: H1: small juveniles with a shell height of 7.5–12.5 mm; H2: juveniles with a shell height of 15.0–25.0 mm; and H3: adults of 25.0–35.0 mm. (a–f) described the survivals at cold acclimation temperatures treatments (i.e., 12°C–30 days, 9°C–30 days, 6°C–10 days, 3°C–10 days, 0°C–1 days, and −3°C–1 days) respectively

**FIGURE 3:**
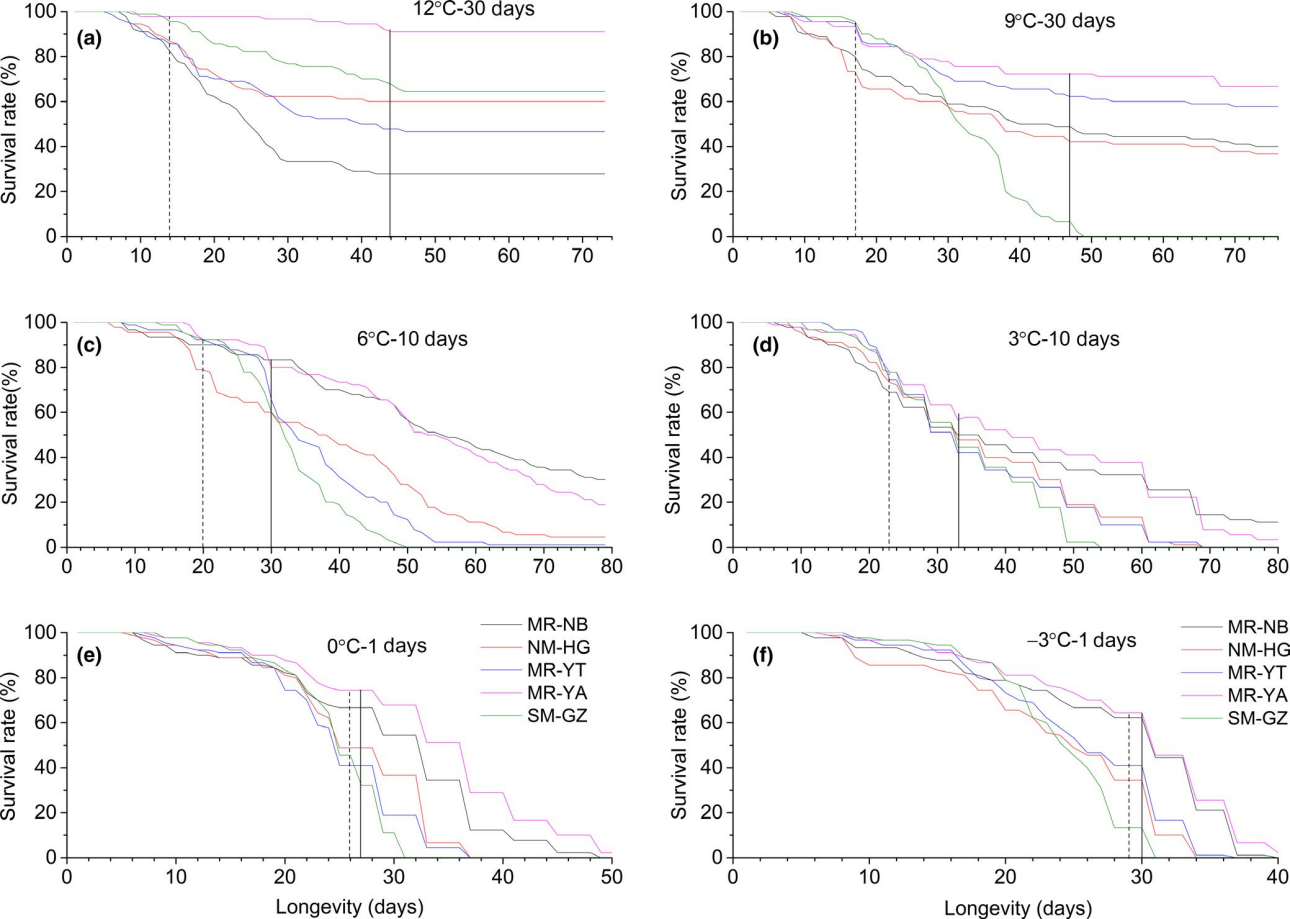
Survival plot for five geographic populations of *P. canaliculata* at each experimental acclimation temperature. (a–f) described the survivals at the six cold acclimation temperatures levels (12, 9, 6, 3, 0, and −3°C) respectively. The five geographic *P. canaliculata* populations were as follows: Guangzhou population in southernmost (SM‐GZ, olive line), three populations of Yingtan (MR‐YT, blue line), Ningbo (MR‐NB, black line), Ya'an (MR‐YA, magenta line) in midrange, and Huanggang population in northernmost (NM‐HG, red line) subtropical zone

When held for 30 days at 12°C, the MR‐YA population had the significantly highest average survival rate (92.2%), and the MR‐NB population had the lowest (27.8%) (Figure 2a). All *P. canaliculata* populations showed constant performance after acclimated to 12°C for 30 days. No other deaths occurred throughout the next month. Compared with the other populations showing rapid decreasing of survival rate, snails from MR‐YA exhibited the most stable survival rate during 60 days of thermal acclimation. Survival rate of the MR‐NB population dropped remarkably at the initial acclimation and declined steadily, exhibiting the most dramatically varying trend in response to stressful temperature. Snails from MR‐YT, NM‐HG, and SM‐GZ had similar survival levels and viability pattern over the acclimation process (Figure 3a).

For 9°C (30 days) and 6°C (10 days) acclimation treatments, populations from MR‐YA, MR‐NB, and MR‐YT (showing the same degree of acclimation) had significantly higher survivorship than the other two populations. *Pomacea canaliculata* from SM‐GZ showed the lowest survival rate (9°C) or the same low level with NM‐HG population (6°C). When held at 9°C, a general duration of more than 60 days was recorded for *P. canaliculata* populations except SM‐GZ population (Figure 2b). Under 6°C, populations from MR‐NB and MR‐YA lived longest (more than 60 days), followed by NM‐HG and MR‐YT populations (Figure 2c). Survival of SM‐GZ population showed drastic decline as time elapsed. Under 9 and 6°C treatments, all individuals of SM‐GZ population died after they acclimated for 32 and 30 days, respectively. Survival of the other four populations exhibited a clear pattern of substantial drop followed by a gradual decrease after being stressed for about 20 days (Figure 3b–c). Under the three temperature acclimation treatments (12, 9, and 6°C), no significant differences occurred between snails of different shell heights. The magnitude of average survival rate in H3 group (25 mm ≤ H ≤ 35 mm) was relatively higher than that in the other two size groups.

Populations did not differ overall in cold resistance and showed similar acclimation responses when exposed to 3°C (10 days). *Pomacea canaliculata* from MR‐YA had a relatively higher survival rate among the studied populations (Figure 2d). *Pomacea canaliculata* from SM‐GZ was the shortest‐lived population. All individuals died after stressed at 3°C for 30 days. MR‐YA and MR‐NB population were the two longest‐lived populations. Their average longevity could reach up to 70 and 65 days under 3°C. NM‐HG and MR‐YT populations had significantly shorter survival time than these two populations, with a 41–44 days longevity (Figure 3d).

Snails with the highest shell height (H3 group, 25 mm ≤ H ≤ 35 mm) obtained no more than 50% survival, significantly lower than the other two size groups. The H3 group of MR‐YT had the lowest survival rate of 53.3%. Under 0 and −3°C acclimation treatments, MR‐YA and MR‐NB populations had significantly higher survival rates than the other *P. canaliculata* populations (Figure 2e–f). No snail from SM‐GZ survived after stressed for 5 days (0°C) and 2 days (−3°C), while survival of MR‐YA population was not impacted until after 26 days at 0°C and 12 days at −3°C. Shell height was detected to have a significant effect on survivorship (Table 2), in which snails with the shortest shell height (H1 group, 5 mm ≤ H ≤ 15 mm) had the lowest survival rates under 0°C (5 days) and −3°C (2 days) conditions. For MR‐YA and MR‐NB populations at 0°C, snails in the H1 group had significantly shorter life spans than those of the two other size groups. Almost all the snails from H1 group of SM‐GZ population were dead after acclimated to −3°C for 1 day, while the H1 group of MR‐YA and MR‐NB population achieved an average of 56.7% and 60.0% survival under the same conditions. When measures of survival rates for each population in varying sizes were combined into a single estimate, all *P. canaliculata* populations survived for a certain amount of time (60 days in this study) under 12°C acclimation. Four populations (not including the SM‐GZ population) exhibited almost the same survival time at 9°C.

**TABLE 2:** Two‐way analyses of variance (ANOVAs) on survival rates of the five *P. canaliculata* populations with location and snail's shell height as fixed effects

Acclimation temperature regime	Location (*df* = 4)		Shell height (*df* = 2)		Location × Shell height (*df* = 8)		Residuals (*df* = 30)
	MS	*F*	MS	*F*	MS	*F*	MS
12°C–30 days	0.518	19.592*	0.025	0.933	0.064	2.424	0.026
9°C–30 days	0.571	14.951*	0.033	0.860	0.024	0.635	0.038
6°C–10 days	0.120	6.685*	0.033	1.815	0.038	2.093	0.018
3°C–10 days	0.019	0.835	0.263	11.485*	0.043	1.862	0.023
0°C–1 day	0.187	9.269*	0.225	11.110*	0.058	2.882	0.020
−3°C–1 day	0.402	22.892*	0.228	13.000*	0.013	0.753	0.018

Note

*df*, degree of freedom; *MS*, mean square; *F*, variance ratio.

* *p* < .05.

For each temperature acclimation treatment, a strong effect of geographic location could be found on overall survivorship of *P. canaliculata* (Table 2)*.* Significant differences were detected among the five geographic populations (except at 3°C for 10 days). Regardless of the acclimation treatment, the MR‐YA population had the highest survival rate and the prolonged survival time (Figure 2). The MR‐NB population had the second‐largest surviving population. During the study period, MR‐NB population's survival duration was at the same level (6 and 3°C) or similar to the MR‐YA population (at 0 and −3°C). At extreme cold (9°C and below), the southern subtropical population of SM‐GZ had the lowest average survival and the shortest longevity.

Cox proportional hazards model results showed that the risk of mortality was significantly influenced by temperature, geographic location, and body size (for the overall model, chi‐square = 270.812, *df* = 13, *p* < .001). The risk of mortality faced by the snail increased significantly at acclimation temperature levels less than 3°C, compared with those at temperature of 12°C. When held at 0 and −3°C, the snail had nearly doubled hazard ratios as that faced at 12°C. Compared with the SM‐GZ population, snails from the other four locations faced less mortality risk, especially for the MR‐NB and the MR‐YA populations. The hazard ratios for snails in these regions (Ya'an and Ningbo) were 0.78 and 0.40 times than that from Guangzhou, respectively. The NM‐HG and MR‐YT populations faced no significant increase in risk. The hazard ratio of 0.712 for juveniles in H2 group means that snails of intermediate size experienced 1.4‐fold decrease in mortality risk compared with those of smaller size (H3 group). Contrastly, adult snail (H1 group) experienced non‐significantly greater mortality risk than small juveniles (Table 3).

**TABLE 3:** Cox proportional hazards model testing for effects of temperature, geographic location, and body size on mortality risk of *Pomacea canaliculata.* Significant effects are shown in bold type (*p* < .05). Estimated regression coefficients *t* (*β*) and its standard error (*SE*), hazard ratios corresponding to each treatment (compared with the control) and its 95% fiducial limits are provided

Variables	*β*	*SE*	Hazard ratio	Sig.	95% confidence interval	
					Lower bound	Upper bound
Acclimation temperature (°C) (control: −3°C)				0.000		
12°C	.643	.102	1.902	**0.000**	1.558	2.322
9°C	.666	.100	1.946	**0.000**	1.599	2.367
6°C	.205	.107	1.227	0.055	0.995	1.513
3°C	−.276	.117	0.759	**0.019**	0.603	0.955
0°C	−.017	.113	0.983	0.880	0.788	1.226
Geographic location (control: SM‐GZ population)				0.000		
MR‐NB	−.249	.089	0.780	**0.005**	0.656	0.928
NM‐HG	−.042	.085	0.959	0.621	0.812	1.133
MR‐YT	−.089	.085	0.915	0.292	0.775	1.080
MR‐YA	−.921	.104	0.398	**0.000**	0.324	0.488
Shell height (mm) (control: H1 group 7.5–12.5 mm)				0.000		
H2 group (15.0–25.0 mm)	−.340	.074	0.712	**0.000**	0.616	0.823
H3 group (25.0–35.0 mm)	.059	.068	1.061	0.386	0.928	1.213

### 3.2 Relationship of survival‐acclimation temperature

**FIGURE 4:**
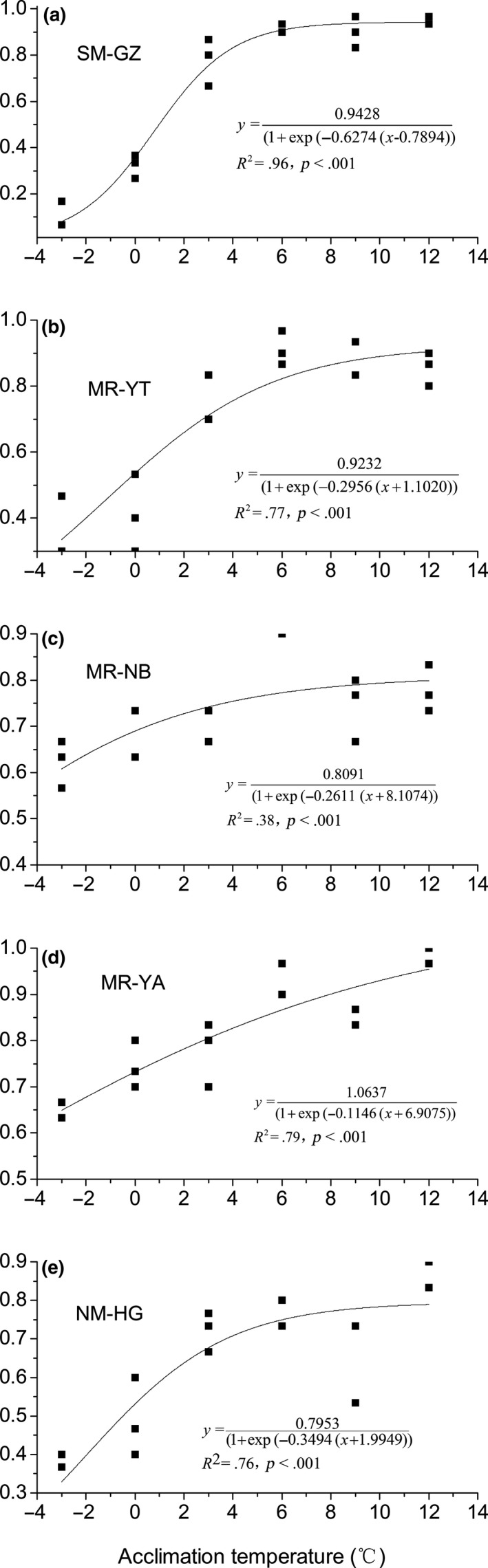
Correlation between acclimation temperature (°C) and corresponding average survival rate (%) for each geographic populations of *P. canaliculata:* (a), (c), and (d) described the relationship for *P. canaliculata* populations from Ningbo (MR‐NB), Yingtan (MR‐YT), and Ya'an (MR‐YA) in midrange subtropical zone, respectively. (b) and (e) described the relationship for *P. canaliculata* populations from Huanggang (NM‐HG) in northernmost and Guangzhou (SM‐GZ) in southernmost subtropical zone. Values are based on survival following a 1 day exposure to the target experimental temperature. Temperature‐dependent survival for each *P. canaliculata* population was estimated with Slogistic function

Generally, survivorship decreased significantly with lower environmental temperatures and exhibited a substantial variability for each *P. canaliculata* population. The effects of low temperature on survivorship of *P. canaliculata* could be described with Slogistic function (Figure 4). Survivorship of all *P. canaliculata* populations showed a positive relationship to acclimation temperatures, with different rates of change. The MR‐YA population had the lowest rate (0.1146) while SM‐GZ population had the highest rate (0.6274). The low temperatures at which 50% of the two populations could survive were −7.95 and 0.98°C, respectively. For the MR‐NB population, 50% of snails could survive at −5.88°C. This estimated threshold was also lower than the other populations. The predicted survival rate of SM‐GZ population closely matched the observed active snails at low temperatures (Figure 4e). The fitted curve showed great fluctuation, especially between low lethal temperature (−3°C) and 6°C, indicating a rapid increase in population surviving gradient. The initial part of the fitted curve showed a substantial increase in population survivorship gradient in the temperature range of −3 to 3°C. Increasing survival rate of the snail dropped down and fell a constant value (100% survive rate) with temperatures over 6°C.

## 4 DISCUSSION

### 4.1 Effects of geographic region and shell height

*Pomacea canaliculata* develops a certain degree of cold tolerance with decreasing temperature (Wada & Matsukura, 2007). The four mid‐ and north subtropical *P. canaliculata* populations reared in shallow water could survive 3°C for more than 40–62 days. Their survival time was 12–26 days, 6–13 days at 0 and −3°C, respectively. The results fell in line with a study (Oya, 1987) in north Kyushu, in which snails kept in storage incubators at 0 and −3°C died within 25 and 3 days, respectively. It also agrees with a study (Matsukura, Tsumuki, Izumi, & Wada, 2009) that found all the snails died of indirect chilling injury within a month regardless of whether they had acclimated to low temperatures. Both previous studies and our results suggest that the survival of overwintering snails in Asia's temperate and subtropical zones were strongly affected by low temperatures below 0°C. It should be noted that previous works on the cold tolerance of *P. canaliculata* were tested at moist or dry condition instead of an aquatic condition. Therefore, comparison of the present data with the previous works is not always effective, which highlights the issue that should be considered during implementation of cold tolerance testing.

Body size of snails affects the survival of *P. canaliculata* during dormancy (Yusa, Wada, et al., 2006). For each experimental *P. canaliculata* population held at 3, 0 and −3°C, individuals with shell height of 15.0–25.0 mm (H2 group, 15.0–25.0 mm) were observed to have significantly higher survival rates than those of H1 (7.5–12.5 mm) or H3 (25.0–35.0 mm) shell height. For those populations held at 12, 9, and 6°C in the acclimation treatments, the snails in H2 still exhibited higher survival rates generally, though differences were not statistically significant. The findings basically coincided with the reports that juveniles of intermediate size (10–20 mm) were more cold‐tolerant than smaller juveniles (< 10 mm) or adults (Syobu et al., 2001; Wada & Matsukura, 2007). At 3°C, snails of the highest shell height (H3 group) had the lowest survivorship, whereas snails of the lowest shell height (H1 group) had the lowest survivorship below 3°C. Therefore, survivorship of the snails may depend on their shell height and the level of low temperature. Smaller juvenile snails seemed tolerate to cold better than larger adults. The findings are consistent with previous reports (Oya, 1987; Syobu et al., 2001) and are supported by field surveys from temperate Japan (Watanabe et al., 2000; Yusa, Sugiura, & Wada, 2006) and southern China (Guo, Luo, Zhang, Luo, & Zhao, 2015), where most overwintering snails were juveniles. One possible explanation for juvenile snails attaining greater tolerance to low temperature than mature ones may lie in the strategy for overwintering. Juvenile snails seemed easier to assure survivorship by decreasing lung ventilation frequency and metabolic rate than adults, based on previous findings (Seuffert & Martín, 2010). However, other involving physiological processes still remain to be solved.

### 4.2 Patterns in responses to acclimation temperatures

For the five studied *P. canaliculata* populations, the MR‐YA population showed the highest viability over the whole thermal range, and the SM‐GZ population exhibited a relative drop in viability at extreme cold (particularly below 3°C). The MR‐YA and MR‐NB populations had the lower fitted temperature at which 50% of the snails were active (*T*_50_) and more smoothly changes of the specific survive rate with temperature, whereas SM‐GZ population had the highest *T*_50_ and wider and faster fluctuations through acclimation temperature regimes (Table 4). Here, we focused on five narrow and well‐defined populations within southern China, though considerable population differences in survivorship as acclimation responses to low temperatures were found, a clear geographic pattern (i.e., decrease of survival rate in descending order of latitude) was not observed. The results agree with comparative studies on responses of insect populations to low temperature (Overgaard, Kearney, & Hoffmann, 2014). Similar findings were also reported in a study on responses to low temperature extremes (without acclimation) in five populations of *P. canaliculata* of China (Dong et al., 2010), in which Kunming population from the middle subtropical zone was more resistant than the other populations. The modeled *T*_50_ did not provide a perfect fit of the observed survivals for mid‐ and north subtropical *P. canaliculata* populations. However, the results captured one dimension of cold adaptation to explain the northward spread of this species and highlighted a need for a more thorough study on cold tolerance for *P. canaliculata* populations, using diverse cold tolerance assays including supercooling point (Matsukura et al., 2009), chill coma temperature (Hazell & Bale, 2011), chill coma recovery time (Sinclair & Roberts, 2005), lethal temperature, and lethal time at cold temperature (EFSA, 2013). It also should be noted that *P. canaliculata* may encounter and respond to many other natural selective regimes (e.g., topography, waters) during its northward expansion, except the stressful low temperature condition. Survivorship of this species may not be affected only by acclimation to temperature. Parallel adaptation of *P. canaliculata* populations may involve gradients such as aridity, desiccation, and air pressure. Acclimation responses to these factors can vary among *P. canaliculata* populations, which suggest that caution is required when detecting population differences in acclimation ability on the basis of a single test of cold tolerance. Unfortunately, few studies were carried out to date to investigate the role of snail acclimation in adaptation to different influential environment factors. The relative contributions of varying selective regimes to habitat suitability for *P. canaliculata* populations still remain unclear.

**TABLE 4:** Parameters (means values, *n* = 3) of the fitted Slogistic curves based on the survivorship (1 day) for each *P. canaliculata* population at each acclimation temperature treatment. Temperature at which 50% of the snails were active (*T*_50_) derived from this function was also shown

Population abbreviation	*a*	*x_c_*	*k*	Reduced Chi‐Sqr	Adj.R‐Square	*T*_50_ (°C)
SM‐GZ	0.9428	0.7894	0.6274	0.0041	0.9629	0.9830
MR‐YT	0.9232	−1.1020	0.2956	0.0116	0.7745	−0.5375
NM‐HG	0.7953	−1.9949	0.3494	0.0089	0.7550	−0.4873
MR‐YA	1.0637	−6.9075	0.1146	0.0030	0.7933	−7.9539
MR‐NB	0.8091	−8.1074	0.2161	0.0064	0.3773	−5.8821

Note

The Slogistic function describing the specific survive rate of change with temperature was: $y=\frac{a}{\left(1+\exp\left(-k\times \left(x-x_{c}\right)\right)\right)}$ where *a* is the maximum value the function can take, the parameter *k* controls how steep the change from the minimum to the maximum value is, *x_c_* is the *x*‐value of the sigmoid's midpoint.

### 4.3 Underlying mechanisms

Studies showed that *P. canaliculata* has great plasticity allowing acclimation to low temperatures. For instance, *P. canaliculata* increases its cold hardiness before winter by acclimation of low weight compounds (e.g., glycerol, glutamine, and carnosine) in its body (Matsukura, Tsumuki, Izumi, & Wada, 2008). The carbohydrate metabolic pathways of cold‐acclimated snails were altered, for which the concentration of glucose in the posterior chamber of the kidney and concentration of glycerol in the digestive gland were significantly enhanced (Matsukura et al., 2009). These findings imply that some functional changes related to cold temperatures were detected in invasive *P. canaliculata* populations, as has been confirmed by our recent study (Guo, Xu, Zhang, Zhao, & Luo, 2014). The biochemical components and concentrations in five *P. canaliculata* populations before and after temperature acclimation also indicated that physiological enhancement may contribute to the range expansion of *P. canaliculata* in China (not published). The SM‐GZ population cannot inhibit the Yangtze River Valley and its southern parts because it lacks adequate winter cold tolerance. The difference in cold tolerance among the *P. canaliculata* populations also confirms the importance for snail organisms of adaptation to local climate. It should be noted that, in contrast with other *P. canaliculata* populations, the similar adaptive plasticity of subtropical SM‐GZ population was unexpected, since *P. canaliculata* was never exposed to subzero temperatures in Guangzhou. This might be the reason for a lack of freeze tolerance in this population.

Previous molecular analyses indicated that Asian *P. canaliculata* populations were the result of multiple introductions from northern Buenos Aires Province, Argentina (34°S–35°S) (Hayes, Joshi, Thiengo, & Cowie, 2008). Recent studies from similar latitudes (33 °N) of temperate Japan populations suggested that cold hardiness could have developed in the native range (Seuffert et al., 2010). *Pomacea canaliculata* also invaded China in the early 1980s, where it has tropical and subtropical natural distributions. High level of genetic diversity and significant genetic differentiation among several sampling locations were observed in mainland China (Xu, Han, Li, et al., 2009). Whether *P. canaliculata* already adapted to cold before it was introduced into China, and whether the life‐history traits (i.e., body size, duration of development, longevity, and productivity) have evolved during their northward extension to cooler areas, is far from clear. Studies to explore whether some of the genetic changes may have facilitated adaptation in two or more colder regions are needed, despite the speculation that cold adaptation seen in *P. canaliculata* might be the consequence of selection pressure exerted by cold winter. Future studies linking physiological, biochemical adjustments, and molecular analysis are also needed to examine in more detail the thermal adaptation and infer the direction of phenotypic or potential genetic variance for acclimation ability of *P. canaliculata* populations.

### 4.4 Management implications

Continuous survival observations were processed over 2 months in this study. All the *P. canaliculata* populations, with average survival of 27.8%‐91.1%, showed a maximum longevity (60 days of test time) at 12°C under aquatic conditions. Smaller proportions (5.6%‐54.4%) from the four *P. canaliculata* populations (not including SM‐GZ population) could survive over the same experimental period at 9°C. It seemed that these proportions of *P. canaliculata* might have survived even longer if the experiment had continued. The current study confirmed that snails could survive over 100 days under 12 and 9°C acclimation treatments (except SM‐GZ population). Though no reproductive activity or accelerated growth was recorded for *P. canaliculata* populations, the snails were observed to continue predating when exposed to 12°C. Overwintering high levels of persistent presence and predation of the snails may impose challenges on local agricultural systems, given the fact that the snails continue to ravage wetland crops and cause potential environmental and biodiversity impacts. For this, rice field–upland field rotation was employed as a practical farming measure to control the snail in southern China. In Guangzhou where *P. canaliculata* can live through the winter successfully, rice and winter potato/ ryegrass rotation system has proved helpful in reducing the density of overwintering snails (Guo et al., 2015; Liu et al., 2014).

## CONFLICT OF INTEREST

The authors declare no conflicts of interests.

## Data Availability

All the experiment datasets (including mitochondrial DNA data of the apple snail) and figures in the article can be accessed at Dryad Digital Repository: https://doi.org/10.5061/dryad.dr7sqv9vf.
